# Association between Intake of Energy and Macronutrients and Memory Impairment Severity in US Older Adults, National Health and Nutrition Examination Survey 2011–2014

**DOI:** 10.3390/nu12113559

**Published:** 2020-11-20

**Authors:** Qinran Liu, Jianjun Guo, Liang Hu, Nicola Veronese, Lee Smith, Lin Yang, Chao Cao

**Affiliations:** 1 Department of Public Health Sciences, University of Miami Miller School of Medicine, Miami, FL 33136, USA; liu.q@miami.edu; 2 Sports and Medicine Integration Center, Capital University of Physical Education and Sports, Beijing 100191, China; 3 Department of Sport and Exercise Science, Zhejiang University, Hangzhou 310027, China; lianghu@zju.edu.cn; 4 Department of Internal Medicine and Geriatrics, University of Palermo, 90133 Palermo, Italy; ilmannato@gmail.com; 5 The Cambridge Centre for Sport and Exercise Sciences, Anglia Ruskin University, Cambridge CB1 1PT, UK; Lee.Smith@anglia.ac.uk; 6 Department of Cancer Epidemiology and Prevention Research, Cancer Care Alberta, Alberta Health Services, Calgary, AB T2S 3C3, Canada; lin.yang@albertahealthservices.ca; 7 Departments of Oncology and Community Health Sciences, Cumming School of Medicine, University of Calgary, Calgary, AB T2N 4N2, Canada; 8 Program in Physical Therapy, Washington University School of Medicine, St Louis, MO 63110, USA; caochao@wustl.edu

**Keywords:** energy intake, memory impairment, carbohydrates, sugar, older adults

## Abstract

Without a cure, dementia affects about 50 million people worldwide. Understanding the effects of dietary habits, a key lifestyle behavior, on memory impairment is critical to inform early behavioral modification to delay further memory loss and progression to dementia. We examined the associations of total energy intake and energy intake from macronutrients with memory impairment among older US adults using data from the nationally representative National Health and Nutrition Examination Survey study 2011–2014. A total of 3623 participants aged ≥60 years were analyzed. Comparing to those with low total energy intake, individuals with high intake were more likely to have severe memory impairment (OR: 1.52, 95% CI: 1.15 to 2.02; *p*_trend_ = 0.005). Specifically, higher energy intake from carbohydrate (OR: 1.59, 95% CI: 1.12 to 2.26) and sugar (OR: 1.54, 95% CI: 1.11 to 2.16) were both significantly associated with the presence of memory impairment. Additionally, higher energy intake from fat, carbohydrate and sugar were significantly associated with more server memory impairment (fat: *p*_trend_ = 0.04; carbohydrate: *p*_trend_ = 0.03; sugar: *p*_trend_ = 0.02). High energy intake, either total or from carbohydrates, fat or sugar, is associated with memory impairment severity in the older US population. No such association was found in energy intake from protein.

## 1. Introduction

Dementia is a syndrome characterized as deterioration in cognitive function, memory loss, and problems controlling emotions [1]. Worldwide, about 50 million people were diagnosed with dementia and there are nearly 10 million new cases every year [2]. The true burden of dementia is likely to be higher due to the lack of a single diagnostic test for dementia and its subtypes [3]. Alzheimer’s disease (AD) is the most common cause of dementia [4], affecting an estimate of 44 million individuals. AD is also the sixth-leading cause of death [5], costing the healthcare system up to $277 billion in the United States in 2018 [6]. Although the disease progression of AD varies from person to person, it is typically associated with a decline in cognitive and functional abilities [7]. Emerging data suggest that memory impairment might be an early sign of AD. Prospective studies found subject memory impairment was commonly reported among individuals years before they developed AD and dementia [8,9]. Even without the presence of dementia, the perception of memory problems is associated with negative outcomes of individual and societal significance. In addition, the severity of memory impairment was negatively associated with quality of life and various health outcomes [10,11,12]. As there is currently no cure for dementia, developing accessible preventive strategies is an urgent but unmet need [13]. Therefore, it is critical to explore the effects of lifestyle factors including dietary habits on memory impairment, as early behavioral modification may delay further memory loss and disease progression.

The important role of nutrition has been recognized in the prevention of cognitive decline, dementia and AD. Observational studies have identified the protective effects of several dietary components, including antioxidants, n-3 polyunsaturated fatty acids, and B vitamins on cognitive function [14]. A meta-analysis also summarized evidence from longitudinal studies and clinical trials and revealed that higher adherence to healthy eating patterns, such as the Mediterranean diet, was associated with better cognitive function and a lower risk of AD [15,16]. Several randomized clinical trials indicated that well-nourished calorie restriction had a myriad of benefits, including metabolic health, aging-associated biomarkers, and quality of life [17,18,19,20], whereas metabolic syndrome negatively impacts cognitive performance and brain structure [21]. However, limited research focuses on the impact of energy intake from each macronutrient (i.e., carbohydrates, protein, fat) on cognitive outcomes. Some observational studies suggested a higher intake of calories was associated with a high risk of developing AD or dementia but reported inconsistent findings on specific energy sources. Specifically, Luchsinger et al. found that a higher intake of calories and fats but not carbohydrates was associated with a higher risk of AD in individuals carrying the apolipoprotein E ∈ 4 [22]. In contrast, Roberts and his colleagues reported caloric intake from carbohydrates and but not fat and protein increased the risk of mild cognitive impairment or dementia [23]. Nevertheless, comprehensive studies are needed to address the effects of total caloric intake and macronutrient intake, on the presence and severity of memory impairment at the population level, as well as within population subgroups defined by several sociodemographic and behavioral factors.

To address these knowledge gaps, we examined the associations of total energy intake and energy intake from carbohydrates, protein, and fat with memory impairment among older US adults using a nationally representative sample.

## 2. Materials and Methods

### 2.1. Study Population

The National Health and Nutrition Examination Survey (NHANES) study is a series of cross-sectional nationally representative health examinations conducted by the National Center for Health Statistics. Since 1999, the NHANES collects data using complex, stratified, multistage, clustered samples to estimate the prevalence of the health, nutritional status and potential disease risk factors among the civilian noninstitutionalized US population in 2-year cycles [24]. Each survey participant completed a written informed consent, a household interview, and a physical examination at a Mobile Examination Center (MEC). We extracted and aggregated data on sociodemographic characteristics, measured weight and height, lifestyle behavior, medical condition among adults aged 65 and older in 2 waves, 2011–2012 and 2013–2014, due to the availability of memory impairment data.

### 2.2. Assessment of Exposure

The NHANES 24-h dietary recall was developed by the National Cancer Institute (NCI) and provided validated information on the amount in grams of each food and beverage consumed during the 24-h period prior to the interview [25]. Additionally, the NHANE dietary interview component, called What We Eat in American, is conducted as a partnership between the U.S. Department of Agriculture (USDA) and the U.S. Department of Health and Human Services (DHHS). Under this partnership, DHHS’ National Center for Health Statistics (NCHS) is responsible for the survey sample design and all aspects of data collection and USDA’s Food Surveys. The first interview was conducted in-person by a trained interviewer in the MEC. The 24-h dietary recall is administered using a proxy interview or an interpreter if needed (e.g., participants who cannot recall their dietary information due to cognitive impairment) [25]. Daily total and nutrient specific energy intake (calories) were extracted from foods and beverages documented in the total Nutrient intakes files, including total calorie intake, total intake of carbohydrate, protein, fat, sugar, saturated fatty acid, monounsaturated fatty acid and polyunsaturated fatty acid. Sex-specific tertile categories were applied for each source of energy intake. The Low group was defined as the first tertile, the moderate group was defined as the second tertile category; and the high group was defined as the third tertile category.

### 2.3. Outcome Measures

Identification of the memory impairment and severity were acquired from the medical condition questionnaire by trained interviewers using the Computer-Assisted Personal Interviewing system [7,26]. Participants were asked “During the past 7 days, how often have you had trouble remembering where you put things like keys or wallet?” The response options included “never”, “about once”, “two or three times”, “nearly every day” and “several times a day”. This ordinal variable was used to reflect memory impairment severity. The participants who responded “never” were categorized as no memory impairment, otherwise as to any memory impairment. This measurement was used in the previous literature to evaluate the early sign of memory impairment (Table 1) [7].

### 2.4. Socio-Demographic Characteristics and Lifestyle Behaviors

Self-reported sociodemographic characteristics included age, sex, race/ethnicity (non-Hispanic white, non-Hispanic black, Hispanic, and others), family income-to-poverty ratio (<1.3 [lowest income], 1.3 ≤ 3.5, ≥3.5 [highest income]), and educational level (less than high school, high school, and above high school) [27,28]. Participants’ weight and height were measured during the physical examination following standard procedures. Body mass index (BMI) was calculated as weight in kilograms divided by height in meters squared and categorize into three groups (<25 kg/m^2^, 25.0–29.9 kg/m^2^, ≥30 kg/m^2^). Leisure-time physical activity status was defined by engaging in no (inactive) or any (active) moderate or vigorous recreational physical activity over the past 30 days [29]. The Healthy Eating Index-2010 (HEI-2010, derived from 24-h dietary recall interviews). HEI-2010 indicates the overall dietary quality with a score ranged from 0 (worst-quality diet) to 100 (best-quality diet) [30].

### 2.5. Chronic Condition

Hypertension was determined by participants receiving a diagnosis from a health professional, or NHANES measured blood pressure ≥130 mm Hg systolic or ≥80 mm Hg diastolic [31]. Hypercholesterolemia was determined by participants receiving a diagnosis from a health professional or NHANES measured total cholesterol level ≥6.2 mmol/L (240 mg/dL) [32]. Cardiovascular disease was identified through participants self-reported ever being diagnosed with conditions such as congestive heart failure, angina, heart attack, or coronary heart disease. Participants were considered as having cancer by self-reported having ever been told by a physician that they had such conditions. Diabetes was defined by self-reporting having been told by a physician that they had diabetes or reporting currently taking insulin to treat diabetes [33].

### 2.6. Statistical Analysis

All analyses followed the NHANES analytical guideline. Survey analysis procedures were used to account for the complex survey design to ensure nationally representative estimates [24]. We conducted a descriptive analysis to assess participants’ characteristics according to whether they have memory impairment. Weighted means (standard error) were calculated for continuous variables, and weighted frequency percentages were calculated for categorical variables. The t-test and chi-square tests were conducted to examine the difference across participants’ characteristics as appropriate.

Then, the associations between different sources of calorie intake (including total energy intake, energy intake from carbohydrate, protein, fat, total sugar, total saturated fatty acid, total monounsaturated fatty acid and total polyunsaturated fatty acid) and the memory impairment (no vs. any) were assessed using weighted logistic regression, respectively Multivariable logistic regression models were adjusted for age, sex, race/ethnicity, education attainment, and family poverty ratio, physical activity, alcohol intake, BMI, body weight, smoking status, hypertension, hypercholesterolemia, family history of diabetes, history of CVD, and history of cancer. In addition, the associations between energy intake (total and nutrient specific) and memory impairment severity using an ordinal variable (“never”, “about once”, “two or three times”, “nearly every day” and “several times a day”) were investigated using multivariable-adjusted ordinal logistic regression models, respectively Only one individual dietary component was included in each regression model.

All statistical analyses were conducted using STATA, version 15.1 (StataCorp, College Station, TX, USA). All statistical significance was set at *p* < 0.05. *p* values were not adjusted for multiple tests and should be interpreted as exploratory analyses.

## 3. Results

A total of 3623 participants aged ≥60 years were included in the analysis. Characteristics of the participants are presented according to memory impairment status in Table 2. Of participants, 20.6% and 7.7% have any kind of memory impairment and late-stage memory impairment, respectively. Female participants (24.6%) had higher prevalence of memory impairment compared to males (15.9%) (*p* < 0.001). Non-Hispanic and Hispanic individuals were more likely to have memory impairment compared to non-Hispanic whites and others (*p* = 0.001). The prevalence of memory impairment was significantly higher among participants with CVD history (25.2% vs. 19.3%, *p* = 0.008). Additionally, participants who were physically inactive (22.7%), had lower education level (<high school: 27.2%), and lower poverty ratio (<1.3: 24.8%) were more likely to have memory impairment comparing to participants who were physically active (17.9%) (*p* = 0.012), had higher education level (high school: 22.8%, >high school: 17.7%, *p* = 0.001), and higher poverty ratio (1/3 ≤ 3.5: 21.2%, ≥3.5: 17.4%, *p* = 0.015).

**Figure 1:**
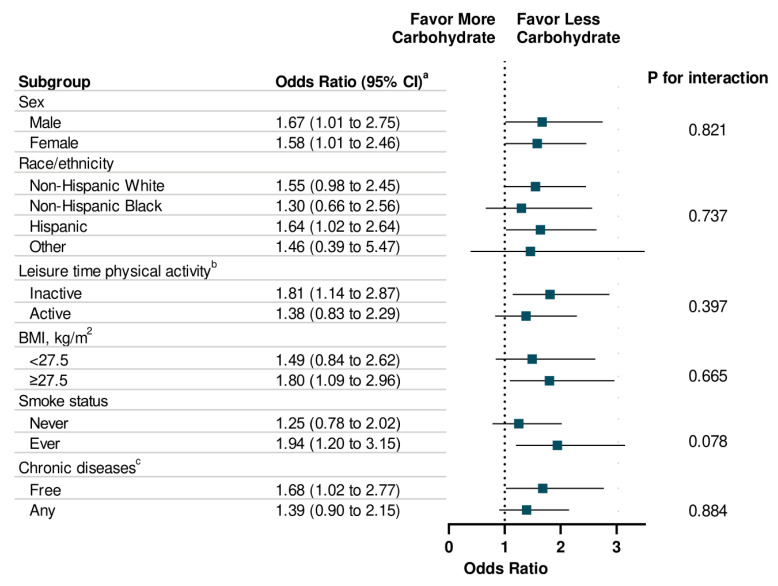
Stratification Analysis on Association Between Energy Intake from Carbohydrate and Memory Impairment Among US Adults ≥60 years, NHANES 2011–2014. ^a^ Multivariable models were adjusted for age, sex, race/ethnicity, education attainment, and family poverty ratio, physical activity, HEI-2010, alcohol intake, BMI, Body Weight, smoking status, hypertension, hypercholesterolemia, family history of diabetes, history of CVD, and history of cancer. ^b^ Leisure-time physical activity level was defined by engaging in no (inactive) or any (active) moderate or vigorous recreational physical activity over the past 30 days. ^c^ Chronic Diseases included diabetes, CVD, and Cancer.

**Figure 2:**
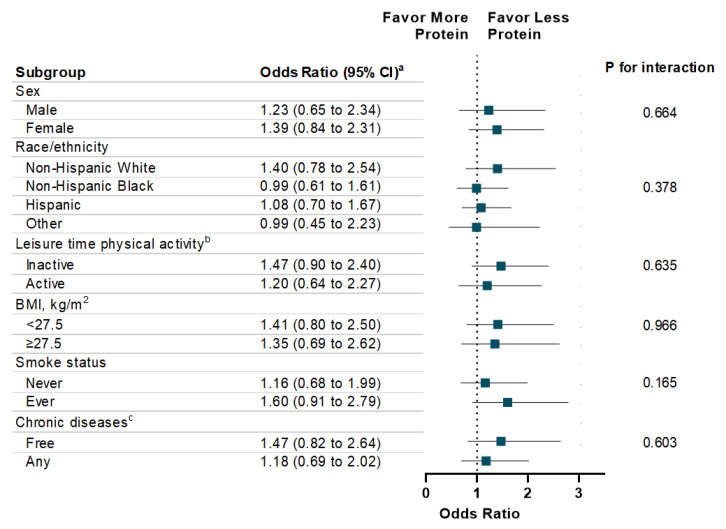
Stratification Analysis on Association Between Energy Intake from Protein and Memory Impairment Among US Adults ≥60 years, NHANES 2011–2014. ^a^ Multivariable models were adjusted for age, sex, race/ethnicity, education attainment, and family poverty ratio, physical activity, HEI-2010, alcohol intake, BMI, Body Weight, smoking status, hypertension, hypercholesterolemia, family history of diabetes, history of CVD, and history of cancer. ^b^ Leisure-time physical activity level was defined by engaging in no (inactive) or any (active) moderate or vigorous recreational physical activity over the past 30 days. ^c^ Chronic Diseases included diabetes, CVD, and Cancer.

**Figure 3:**
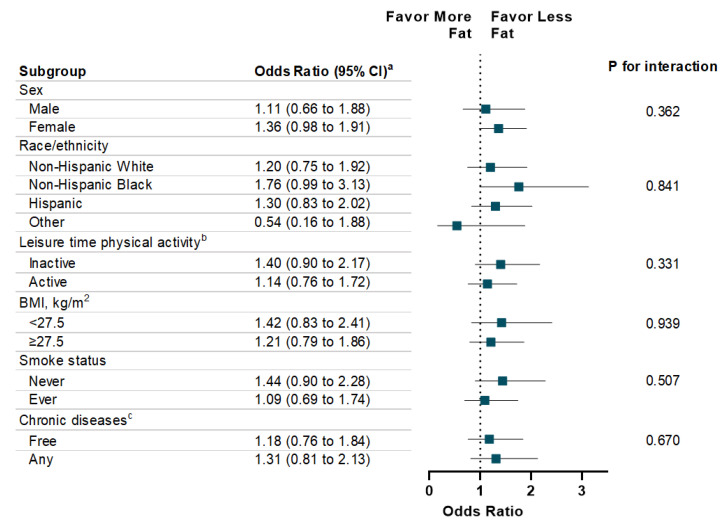
Stratification Analysis on Association Between Energy Intake from Fat and Memory Impairment Among US Adults ≥60 years, NHANES 2011–2014. ^a^ Multivariable models were adjusted for age, sex, race/ethnicity, education attainment, and family poverty ratio, physical activity, HEI-2010, alcohol intake, BMI, Body Weight, smoking status, hypertension, hypercholesterolemia, family history of diabetes, history of CVD, and history of cancer. ^b^ Leisure-time physical activity level was defined by engaging in no (inactive) or any (active) moderate or vigorous recreational physical activity over the past 30 days. ^c^ Chronic Diseases included diabetes, CVD, and Cancer.

The associations between energy intake (total and macronutrient-specific) and memory impairment are shown in Table 3. Individuals with high total energy intake were more likely to report severe memory impairment compared to those with low total energy intake (OR: 1.52, 95% CI: 1.15 to 2.12; *p* for trend = 0.005). With respect to specific macronutrients, a dose-response relationship was observed between energy intake from carbohydrates and the presence (*p* for trend = 0.01) and the severity level (*p* for trend = 0.03) of memory impairment. Additionally, high energy intake from fat is associated with memory impairment severity (*p* for trend = 0.04). There was no statistically significant association observed between energy intake from protein and memory impairment. In addition, high energy intake from sugar intake was significantly associated with memory impairment (OR: 1.54, 95% CI: 1.11 to 2.16) and severity of memory impairment (OR: 1.52, 95% CI: 1.12 to 2.09) (Table 4). In addition, high energy intake from total saturated fatty acid was associated with memory impairment severity (*p* for trend = 0.02). Finally, the associations of energy intake from carbohydrate, protein, and fat with memory impairment were consistent across each subgroup, such as sex, race/ethnicity, physical activity, weight status, smoke status, and chronic diseases (Figure 1, Figure 2 and Figure 3).

## 4. Discussion

In this large representative sample of US older adults, higher total energy intake was associated with higher memory impairment severity, after adjusting for an array of potential confounders including sociodemographic characteristics, lifestyle factors, and chronic conditions. Specifically, energy intake from carbohydrate was associated with memory impairment. This association kept consistent across sex, race/ethnicity, physical activity level, weight status, smoke status, and chronic diseases. However, energy intake from neither protein nor fat was related to memory impairment. Further exploratory results suggested that more energy intake from sugar was significantly associated with a higher likelihood of memory impairment.

Our study extended the previous evidence on the association of total and macronutrient-specific energy intake with memory impairment at the population level. Findings from the present analyses were in line with previous studies that investigated a limited number of macronutrients in a smaller sample. Specifically, a prospective study followed up with 980 individuals free of dementia at baseline for 4 years. Comparing to lower energy intake, higher calorie and fat intake were associated with a 2-fold increased risk of AD among individuals with the apolipoprotein E ∈4 allele but not among those without the apolipoprotein E ∈4 allele [22]. Another prospective cohort of 937 elderly adults with a median age of 79.5 found a dietary pattern with relatively high caloric intake from carbohydrates and low caloric intake from fat and proteins was linked with a higher risk of mild cognitive impairment or dementia [23]. A cross-sectional study found that the dietary pattern with a high percentage of energy intake from fat and protein, and low-energy intake from carbohydrate was associated with impaired cognitive function in 661 Chinese young adults [34]. The present association between sugar intake and memory impairment agreed with previous findings that habitual sugar intake appeared to be associated with poor cognitive function [35]. However, evidence on the effects of energy intake on memory impairment is lacking among adults at early stages, because previous studies enrolled most participants at very advanced ages (around 80 years). Our findings extended previous evidence to the early age stage of older adults (≥60 years) at the population level. Meanwhile, our stratification analyses indicated that the present association was consistent across different subpopulation, such as sex, race/ethnicity, physical activity level, weight status, and chronic diseases.

Several potential biological pathways could explain the negative association between energy intake and memory function. Animal studies found that a high-calorie diet could induce the activation of an inflammatory response (e.g., increased in reactive astrocytes and interleukin1-β) and oxidative stress (e.g., reactive oxygen species and lipid peroxidation), as well as reducing the number of neurons in the temporal cortex and hippocampus, which contribute to neurodegeneration and memory impairments [18]. A theoretical model was proposed that excessive energy intake leads to increased oxidative stress, impaired protein degradation, and elevated inflammation, which may negatively impact synaptic plasticity and neurogenesis and cause cognitive deficits and AD [36]. Specifically, some research indicated that high sugar consumption causes inflammation in the brain, leading to hippocampal-dependent memory problems [37], whereas such negative effects may be reversed by reducing sugar intake and supplementing with omega-3 fatty acids and curcumin [38]. In addition, excess caloric intake could alter the brain’s reward system and result in a progressive addiction to foods that are low-nutrient but rich in sugar. Lenoir and colleagues demonstrated that intense sweetness could surpass cocaine reward, even in drug-sensitized and -addicted individuals [39]. Added sugar intake was associated with a wide range of health problems, such as cardiovascular diseases and diabetes, which are both linked to neurodegeneration and cognitive decline [40].

The 2015–2020 Dietary Guideline for Americans encourages individuals to adhere to a healthy eating pattern across the lifespan. The guideline highlighted the importance of consuming less than 10% of total calories from added sugars each day and reducing the consumption of sugar-sweetened beverages such as soft drinks, which has contributed to the high prevalence of obesity in the United States [41]. Our findings provided additional evidence supporting the benefits of a healthy diet in cognition and memory function. Furthermore, further research is needed to confirm the causal relationship between energy intake and memory impairment.

A clear strength of this study is the use of a large representative sample of the older US population to generalize the findings at the population level. This study is not without limitations. First, the 24-h dietary call interview may cause recall bias. However, data on participants who cannot recall their dietary information were to be collected from their proxies (e.g., caregivers). In addition, the dietary data were comprehensively reviewed by the trained NHANES staff to avoid potential inaccurate data. Second, the memory impairment was not diagnosed by an expert evaluating the Clinical Dementia Rating Scale. However, in the present study, using an early indicator of memory impairment based on structured questions during the in-person interview may provide valuable evidence on preventive strategies. Third, due to the nature of the study design, the causality cannot be determined. Indeed, longitudinal studies using repeated-measurement and clinical trials are further needed to investigate the effect of macronutrient energy intake on memory deterioration changes in a long-term period.

## 5. Conclusions

In conclusion, energy intake is significantly associated with worsened memory impairment severity in the older US population. In particular, the energy intake from carbohydrates and fat, but not protein, is linked to memory impairment. These findings provide evidence on the potential cognitive benefits of healthy eating patterns for preventing AD and dementia, and highlight the need of developing strategies for promoting a healthy diet among older adults, especially for those at risk of memory impairment.

## Figures and Tables

**Table 1:** The Severity of Memory Impairment Using NHANES 2011–2014 Memory Question.

Value Description	Memory Impairment Severity Classification
Never	None
About once	Early-stage
Two or three times	
Nearly every day	Late-stage
Several times a day	

**Table 2:** Characteristics of the US Adults ≥60 years According to Memory Impairment, NHANES 2011–2014 ^a^.

	Memory Impairment		*p* Value
	No	Any	
N	2802	821	
Weighted N	47,243,167	12,303,530	
Age, y	69.2 (0.3)	71.5 (0.4)	<0.001
Sex			
Male	84.1	15.9	<0.001
Female	75.4	24.6	
Race/ethnicity			
Non-Hispanic white	80.1	19.9	0.001
Non-Hispanic black	79.6	20.4	
Hispanic	70.4	29.6	
Other	80.6	19.4	
Family poverty ratio			
<1.3	75.2	24.8	0.019
1.3 ≤ 3.5	78.9	21.2	
≤3.5	82.6	17.4	
Education			
<High school	72.8	27.2	0.001
High school	77.2	22.8	
>High school	82.3	17.7	
Body mass index ^b^, kg/m^2^			
<25	76.1	23.9	0.106
25 ≤ 30	81.0	19.0	
≥30	80.5	19.5	
Leisure-time physical activity ^c^			
Inactive	77.3	22.7	0.012
Active	82.1	17.9	
Cardiovascular Disease			
No	80.7	19.3	0.008
Yes	74.8	25.2	
Cancer			
No	79.3	20.7	0.873
Yes	79.6	20.4	
Diabetes			
No	79.8	20.2	0.379
Yes	77.6	22.4	
Healthy Eating Index-2010	58.7 (0.5)	57.9 (0.9)	0.348

^a^ All estimates were weighted to be nationally representative. ^b^ Weight status was defined by body mass index (BMI = weight(kg)/height(m)^2^). ^c^ Leisure-time physical activity level was defined by engaging in no (inactive) or any (active) moderate or vigorous recreational physical activity over the past 30 days.

**Table 3:** Multivariable-adjusted Association of Source Specific Energy Intake and Memory Severity Impairment Among US Adults ≥60 years, NHANES 2011–2014 ^a^.

	Source of Calorie Intake ^b^			
	Total	Carbohydrate	Protein	Fat
**Memory Impairment ^c^**				
**Energy Intake**				
Low	1 [Reference]	1 [Reference]	1 [Reference]	1 [Reference]
Moderate	1.10 (0.79 to 1.52)	1.34 (0.98 to 1.85)	1.03 (0.71 to 1.50)	1.23 (0.86 to 1.77)
High	1.46 (1.00 to 2.12)	1.59 (1.12 to 2.26)	1.35 (0.88 to 2.06)	1.26 (0.90 to 1.76)
*p* for trend	0.055	0.01	0.15	0.17
**Memory Impairment Severity ^d^**				
**Energy Intake**				
Low	1 [Reference]	1 [Reference]	1 [Reference]	1 [Reference]
Moderate	1.19 (0.95 to 1.49)	1.16 (0.88 to 1.54)	1.10 (0.78 to 1.54)	1.16 (0.84 to 1.59)
High	1.52 (1.15 to 2.02)	1.40 (1.04 to 1.88)	1.39 (0.95 to 2.02)	1.33 (1.01 to 1.76)
*p* for trend	0.005	0.03	0.08	0.04

^a^ Multivariable models were adjusted for age, sex, race/ethnicity, education attainment, and family poverty ratio, physical activity, Healthy Eating Index-2010, alcohol intake, BMI, Body Weight, smoking status, hypertension, hypercholesterolemia, family history of diabetes, history of CVD, and history of cancer. ^b^ Sex-specific tertile categories were applied for each source of energy intake. The Low group was defined as the first tertile, the moderate group was defined as the second tertile category; and the high group was defined as the third tertile category. Only one source of energy intake was included in each model. ^c^ Binary outcome (No vs. Any memory Impairment) was used in the multivariable-adjusted logistic regression models. ^d^ Ordinal outcome (“never”, “about once”, “two or three times”, “nearly every day” and “several times a day”) was used in the multivariable-adjusted ordinal logistic regression models.

**Table 4:** Multivariable-adjusted Association of Source-Specific Energy Intake and Memory Impairment Among US Adults ≥60 years, NHANES 2011–2014 ^a^.

	Source of Calorie Intake ^b^			
	Sugar	Total Saturated Fatty Acid	Total Monounsaturated Fatty Acid	Total Polyunsaturated Fatty Acid
**Memory Impairment ^c^**				
**Energy Intake**				
Low	1 [Reference]	1 [Reference]	1 [Reference]	1 [Reference]
Moderate	1.43 (1.04 to 1.96)	1.35 (0.95 to 1.92)	1.14 (0.85 to 1.54)	1.15 (0.80 to 1.64)
High	1.54 (1.06 to 2.24)	1.31 (0.97 to 1.78)	1.23 (0.91 to 1.67)	1.34 (0.94 to 1.92)
*p* for trend	0.02	0.07	0.17	0.09
**Memory Impairment Severity ^d^**				
**Energy Intake**				
Low	1 [Reference]	1 [Reference]	1 [Reference]	1 [Reference]
Moderate	1.37 (1.04 to 1.80)	1.13 (0.85 to 1.49)	1.20 (0.93 to 1.55)	1.14 (0.86 to 1.50)
High	1.42 (1.05 to 1.92)	1.40 (1.07 to 1.84)	1.31 (0.98 to 1.75)	1.26 (0.96 to 1.66)
*p* for trend	0.02	0.02	0.07	0.10

^a^ Multivariable models were adjusted for age, sex, race/ethnicity, education attainment, and family poverty ratio, physical activity, Healthy Eating Index-2010, alcohol intake, BMI, Body Weight, smoking status, hypertension, hypercholesterolemia, family history of diabetes, history of CVD, and history of cancer. ^b^ Sex-specific tertile categories were applied for each source of energy intake. The Low group was defined as the first tertile, the moderate group was defined as the second tertile category; and the high group was defined as the third tertile category. Only one source of energy intake was included in each model. ^c^ Binary outcome (No vs. Any memory Impairment) was used in the multivariable-adjusted logistic regression models. ^d^ Ordinal outcome (“never”, “about once”, “two or three times”, “nearly every day” and “several times a day”) was used in the multivariable-adjusted ordinal logistic regression models.

## References

[1] Center for Disease Control and Prevention What Is Dementia?. https://www.cdc.gov/aging/dementia/index.html.

[2] Alzheimer’s Association (2020). 2020 Alzheimer’s disease facts and figures. Alzheimer’s Dement..

[3] Rossor M.N., Fox N.C., Mummery C.J., Schott J.M., Warren J.D. (2010). The diagnosis of young-onset dementia. Lancet Neurol..

[4] World Health Organization Dementia. https://www.who.int/news-room/fact-sheets/detail/dementia.

[5] Center for Disease Control and Prevention Leading Causes of Death. https://www.cdc.gov/nchs/fastats/leading-causes-of-death.htm.

[6] Alzheimer’s Association (2018). 2018 Alzheimer’s disease facts and figures. Alzheimer’s Dement..

[7] Aigbogun M.S., Stellhorn R., Krasa H., Kostic D. (2017). Severity of memory impairment in the elderly: Association with health care resource use and functional limitations in the United States. Alzheimer’s Dement..

[8] Jessen F., Wiese B., Bachmann C., Eifflaender-Gorfer S., Haller F., Kölsch H., Luck T., Mösch E., van den Bussche H., Wagner M. (2010). Prediction of Dementia by Subjective Memory Impairment: Effects of Severity and Temporal Association With Cognitive Impairment. Arch. Gen. Psychiatry.

[9] Mitchell A.J., Beaumont H., Ferguson D., Yadegarfar M., Stubbs B. (2014). Risk of dementia and mild cognitive impairment in older people with subjective memory complaints: Meta-analysis. Acta Psychiatr. Scand..

[10] Reisberg B., Shulman M.B., Torossian C., Leng L., Zhu W. (2010). Outcome over seven years of healthy adults with and without subjective cognitive impairment. Alzheimer’s Dement..

[11] Pressler S.J., Subramanian U., Kareken D., Perkins S.M., Gradus-Pizlo I., Sauvé M.J., Ding Y., Kim J., Sloan R., Jaynes H. (2010). Cognitive deficits and health-related quality of life in chronic heart failure. J. Cardiovasc. Nurs..

[12] Pan C.-W., Wang X., Ma Q., Sun H.-P., Xu Y., Wang P. (2015). Cognitive dysfunction and health-related quality of life among older Chinese. Sci. Rep..

[13] Cummings J., Aisen P.S., DuBois B., Frölich L., Jack C.R., Jones R.W., Morris J.C., Raskin J., Dowsett S.A., Scheltens P. (2016). Drug development in Alzheimer’s disease: The path to 2025. Alzheimer’s Res. Ther..

[14] Smith P.J., Blumenthal J.A. (2016). Dietary Factors and Cognitive Decline. J. Prev. Alzheimers Dis..

[15] Van den Brink A.C., Brouwer-Brolsma E.M., Berendsen A.A.M., van de Rest O. (2019). The Mediterranean, Dietary Approaches to Stop Hypertension (DASH), and Mediterranean-DASH Intervention for Neurodegenerative Delay (MIND) Diets Are Associated with Less Cognitive Decline and a Lower Risk of Alzheimer’s Disease—A Review. Adv. Nutr. (Bethesda Md.).

[16] Smyth A., Dehghan M., Donnell M., Anderson C., Teo K., Gao P., Sleight P., Dagenais G., Probstfield J.L., Mente A. (2015). Healthy eating and reduced risk of cognitive decline. Neurology.

[17] Xu B.-L., Wang R., Ma L.-N., Dong W., Zhao Z.-W., Zhang J.-S., Wang Y.-L., Zhang X. (2015). Effects of Caloric Intake on Learning and Memory Function in Juvenile C57BL/6J Mice. BioMed Res. Int..

[18] Treviño S., Aguilar-Alonso P., Flores Hernandez J.A., Brambila E., Guevara J., Flores G., Lopez-Lopez G., Muñoz-Arenas G., Morales-Medina J.C., Toxqui V. (2015). A high calorie diet causes memory loss, metabolic syndrome and oxidative stress into hippocampus and temporal cortex of rats. Synapse.

[19] Redman L.M., Smith S.R., Burton J.H., Martin C.K., Il’yasova D., Ravussin E. (2018). Metabolic Slowing and Reduced Oxidative Damage with Sustained Caloric Restriction Support the Rate of Living and Oxidative Damage Theories of Aging. Cell Metab..

[20] Most J., Tosti V., Redman L.M., Fontana L. (2017). Calorie restriction in humans: An update. Ageing Res. Rev..

[21] Yates K.F., Sweat V., Yau P.L., Turchiano M.M., Convit A. (2012). Impact of metabolic syndrome on cognition and brain: A selected review of the literature. Arterioscler. Thromb. Vasc. Biol..

[22] Luchsinger J.A., Tang M.-X., Shea S., Mayeux R. (2002). Caloric Intake and the Risk of Alzheimer Disease. Arch. Neurol..

[23] Roberts R.O., Roberts L.A., Geda Y.E., Cha R.H., Pankratz V.S., O’Connor H.M., Knopman D.S., Petersen R.C. (2012). Relative intake of macronutrients impacts risk of mild cognitive impairment or dementia. J. Alzheimers Dis..

[24] Cao C., Liu Q., Abufaraj M., Han Y., Xu T., Waldhoer T., Shariat S.F., Li S., Yang L., Smith L. (2020). Regular Coffee Consumption Is Associated with Lower Regional Adiposity Measured by DXA among US Women. J. Nutr..

[25] Raper N. (2004). An overview of USDA’s Dietary Intake Data System. J. Food Compos. Anal..

[26] Centers for Disease Control and Prevention NHANES 2013-2014 Questionnaire Data Overview. https://wwwn.cdc.gov/nchs/nhanes/ContinuousNhanes/OverviewQuex.aspx?BeginYear=2013.

[27] Yang L., Cao C., Kantor E.D., Nguyen L.H., Zheng X., Park Y., Giovannucci E.L., Matthews C.E., Colditz G.A., Cao Y. (2019). Trends in Sedentary Behavior Among the US Population, 2001–2016. JAMA.

[28] Cao C., Hu L., Xu T., Liu Q., Koyanagi A., Yang L., Carvalho A.F., Cavazos-Rehg P.A., Smith L. (2020). Prevalence, correlates and misperception of depression symptoms in the United States, NHANES 2015–2018. J. Affect. Disord..

[29] Roberts J., Liu Q., Cao C., Jackson S.E., Zong X., Meyer G.A., Yang L., Cade W.T., Zheng X., López-Sánchez G.F. (2020). Association of Hot Tea Consumption with Regional Adiposity Measured by Dual-Energy X-ray Absorptiometry in NHANES 2003–2006. Obesity.

[30] Guenther P.M., Casavale K.O., Reedy J., Kirkpatrick S.I., Hiza H.A., Kuczynski K.J., Kahle L.L., Krebs-Smith S.M. (2013). Update of the Healthy Eating Index: HEI-2010. J. Acad. Nutr. Diet..

[31] Whelton Paul K., Carey Robert M., Aronow Wilbert S., Casey Donald E., Collins Karen J., Dennison Himmelfarb C., DePalma Sondra M., Gidding S., Jamerson Kenneth A., Jones Daniel W. (2018). 2017 ACC/AHA/AAPA/ABC/ACPM/AGS/APhA/ASH/ASPC/NMA/PCNA Guideline for the Prevention, Detection, Evaluation, and Management of High Blood Pressure in Adults: A Report of the American College of Cardiology/American Heart Association Task Force on Clinical Practice Guidelines. Hypertension.

[32] Gregg E.W., Cheng Y.J., Cadwell B.L., Imperatore G., Williams D.E., Flegal K.M., Narayan K.M., Williamson D.F. (2005). Secular trends in cardiovascular disease risk factors according to body mass index in US adults. JAMA.

[33] Cao C., Yang L., Cade W.T., Racette S.B., Park Y., Cao Y., Friedenreich C.M., Hamer M., Stamatakis E., Smith L. (2020). Cardiorespiratory Fitness Is Associated with Early Death Among Healthy Young and Middle-aged Baby Boomers and Generation Xers. Am. J. Med..

[34] Ding B., Xiao R., Ma W., Zhao L., Bi Y., Zhang Y. (2018). The association between macronutrient intake and cognition in individuals aged under 65 in China: A cross-sectional study. BMJ Open.

[35] Chong C.P., Shahar S., Haron H., Din N.C. (2019). Habitual sugar intake and cognitive impairment among multi-ethnic Malaysian older adults. Clin. Interv. Aging.

[36] Mattson M.P. (2010). The impact of dietary energy intake on cognitive aging. Front. Aging Neurosci..

[37] Beilharz J.E., Maniam J., Morris M.J. (2016). Short-term exposure to a diet high in fat and sugar, or liquid sugar, selectively impairs hippocampal-dependent memory, with differential impacts on inflammation. Behav. Brain Res..

[38] Beilharz J.E., Maniam J., Morris M.J. (2015). Diet-Induced Cognitive Deficits: The Role of Fat and Sugar, Potential Mechanisms and Nutritional Interventions. Nutrients.

[39] Lenoir M., Serre F., Cantin L., Ahmed S.H. (2007). Intense Sweetness Surpasses Cocaine Reward. PLoS ONE.

[40] Zilliox L.A., Chadrasekaran K., Kwan J.Y., Russell J.W. (2016). Diabetes and Cognitive Impairment. Curr. Diabetes Rep..

[41] US Department of Health and Human Services, US Department of Agriculture (2015). 2015-2020 Dietary Guidelines for Americans.

